# Prognostic Signatures of Metabolic Genes and Metabolism-Related Long Non-coding RNAs Accurately Predict Overall Survival for Osteosarcoma Patients

**DOI:** 10.3389/fcell.2021.644220

**Published:** 2021-02-23

**Authors:** Gong Chao-yang, Tang Rong, Shi Yong-qiang, Liu Tai-cong, Zhou Kai-sheng, Nan Wei, Zhang Hai-hong

**Affiliations:** ^1^Lanzhou University Second Hospital, Lanzhou, China; ^2^Orthopaedics Key Laboratory of Gansu Province, Lanzhou, China; ^3^Department of Anesthesiology, Lanzhou University Second Hospital, Lanzhou, China

**Keywords:** metabolism, lncRNAs, osteosarcoma, signatures, prognostic

## Abstract

In this study, we identified eight survival-related metabolic genes in differentially expressed metabolic genes by univariate Cox regression analysis based on the therapeutically applicable research to generate effective treatments (*n* = 84) data set and genotype tissue expression data set (*n* = 396). We also constructed a six metabolic gene signature to predict the overall survival of osteosarcoma (OS) patients using least absolute shrinkage and selection operator (Lasso) Cox regression analysis. Our results show that the six metabolic gene signature showed good performance in predicting survival of OS patients and was also an independent prognostic factor. Stratified correlation analysis showed that the metabolic gene signature accurately predicted survival outcomes in high-risk and low-risk OS patients. The six metabolic gene signature was also verified to perform well in predicting survival of OS patients in an independent cohort (GSE21257). Then, using univariate Cox regression and Lasso Cox regression analyses, we identified an eight metabolism-related long noncoding RNA (lncRNA) signature that accurately predicts overall survival of OS patients. Gene set variation analysis showed that the apical surface and bile acid metabolism, epithelial mesenchymal transition, and P53 pathway were activated in the high-risk group based on the eight metabolism-related lncRNA signature. Furthermore, we constructed a competing endogenous RNA (ceRNA) network and conducted immunization score analysis based on the eight metabolism-related lncRNA signature. These results showed that the six metabolic gene signature and eight metabolism-related lncRNA signature have good performance in predicting the survival outcomes of OS patients.

## Introduction

Osteosarcoma (OS) is a primary malignant bone cancer and commonly occurs in adolescents and children. The overall annual incidence of OS is 3.4 million worldwide (Mirabello et al., 2009; Pingping et al., 2019; Czarnecka et al., 2020; Mirabello et al., 2020). As a malignant tumor, OS typically occurs in the metaphysis of the long bones, such as the distal femur (43%), proximal tibia (23%), or humerus (10%) (Isakoff et al., 2015). Previous reports suggest that the 5-year survival rate of patients with nonmetastatic OS is 70–75%, but the long-term survival rate of metastatic OS patients is only 30% (Anwar et al., 2020). In addition, multidrug resistance is a major challenge in OS treatment (Strauss et al., 2010). Hence, there is an urgent need to identify novel targets and biomarkers for the diagnosis and prognosis of OS to improve the survival rate of OS patients.

Cancer metabolism is one of the oldest areas of research in cancer biology, and targeting metabolism has been an effective cancer treatment modality for decades (Cairns et al., 2011; Luengo et al., 2017). An increasing amount of evidence shows that changes in cell metabolism contribute to cancer development and progression (Vander Heiden and DeBerardinis, 2017; Kreuzaler et al., 2020). Additionally, several studies show that the tumor suppressor p53, *MYC* oncogene, pyruvate kinase isozymes M2 (PKM2), and hypoxia-inducible factor 1(HIF1) regulate cancer metabolism and are involved in the prognosis of cancers (Denko, 2008; Dayton et al., 2016; Gomes et al., 2018; Khan et al., 2020). Long noncoding RNAs (lncRNAs) are noncoding RNAs more than 200 nucleotides in length and play important roles in transcriptional regulation, epigenetic gene regulation, and disease (Mercer et al., 2009; Kumar and Goyal, 2017). The main difference between lncRNAs and mRNAs is that lncRNAs lack reading frames encoding proteins (Dinger et al., 2011). However, investigations using advanced molecular techniques suggest that some lncRNAs contain short open reading frames (sORFs) and can interact with ribosomes to encode proteins (Chen et al., 2020; Vergara et al., 2020). Recent studies show that lncRNAs play important roles in glucose, protein, lipid, and nucleic acid metabolism by directly or indirectly targeting enzymes and oncogenic signaling pathways (Fan et al., 2017; Zeng et al., 2018; Lu et al., 2019). Tang et al. (2019) found that lncRNA GLCC1 promotes carcinogenesis and glucose metabolism by stabilizing c-Myc, resulting in poor prognosis in colorectal cancer. Another study showed that lncRNA ANRIL is involved in regulating AML development by modulating the glucose metabolism pathway of AdipoR1/AMPK/SIRT1 (Sun et al., 2018). Although many metabolic biomarkers have been identified for the diagnosis and prognosis of human cancers, research on metabolic biomarkers for the prognosis of OS is limited. Therefore, in this study, we sought to identify novel metabolic signatures that are related to the diagnosis and prognosis of OS patients.

In this study, we identified a six metabolic gene signature using bioinformatics that shows good performance in predicting survival of OS patients. Stratified correlation analysis shows that the metabolic gene signature accurately predicted survival outcomes in both high- and low-risk OS patients. The six metabolic gene signature was also verified to perform well in predicting survival of OS patients in an independent data set (GSE21257). Furthermore, we also identified an eight metabolism-related lncRNA signature that is related to overall survival in OS and shows good performance in predicting overall survival of OS. Gene set variation analysis (GSVA) revealed that multiple metabolic processes and signaling pathways were significantly enriched in the high-risk groups. Finally, we constructed a competing endogenous RNA (ceRNA) network based on the eight metabolism-related lncRNA signature and analyzed the immunization scores in high- and low-risk OS patients. Our results suggest that the six metabolic gene signature and eight metabolism-related lncRNA signature identified show robust performance in predicting the survival outcomes of OS patients.

## Materials and Methods

### Data Downloading and Processing

A total of 944 metabolic genes were extracted from metabolism-related Kyoto encyclopedia of genes and genomes (KEGG) pathways. RNA sequence data and clinical information on the OS patients were downloaded from the therapeutically applicable research to generate effective treatments (TARGET^1^). Transcriptome data of normal human tissues were downloaded from the genotype tissue expression (GTEx^2^) database. Expression profiling and clinical information on a test independent data set (GSE21257) was downloaded from the Gene Expression Omnibus (GEO^3^). The clinical characteristics of all OS patients in the two data sets are listed in Table 1. The R software^4^ sva (Li et al., 2018) package was used to merge the raw data of the two sets (TARGET OS *n* = 84 and GTEx OS *n* = 396) and eliminate batch-to-batch differences.

**TABLE 1:** Clinical data for all patients.

Variables	Groups	Training set (TARGET) *(n* = 84)	Test set1 (GSE21257) (*n* = 53)
		Patients no. (%)	Patients no. (%)
Age	≤18	68 (81.0)	39 (73.6)
	>18	16 (19.0)	14 (26.4)
Gender	Female	36 (42.9)	19 (35.8)
	Male	48 (57.1)	34 (64.2)
Metastatic	Metastatic	21 (25.0)	34 (64.2)
	Non-metastatic	63 (75.0)	19 (35.8)
Histologic response	Stage 1/2	18 (21.4)	29 (54.7)
	Stage 3/4	16 (19.0)	18 (34.0)
Vital status	Alive	55 (65.5)	30 (56.6)
	Dead	29 (34.5)	23 (43.4)

### Function Annotation

A total of 2,282 differentially expressed genes were selected based on a cutoff value of | log_2_FC | > 1 and a *P* value less than 0.05 in OS tissue and normal muscle tissue using the Limma (Ritchie et al., 2015) package, and 64 differentially expressed genes were screened from the 2,282 differentially expressed genes. Next, we conducted gene ontology (GO) and KEGG enrichment analysis of the 64 differentially expressed metabolic genes using the clusterProfiler (Kanehisa et al., 2017) package, established a protein–protein interaction (PPI) network, and identified 10 hub metabolic genes using the Search Tool for the Retrieval of Interacting Genes online tool (STRING^5^) and Cytoscape (Shannon et al., 2003).

### Identification and Construction of Prognostic Signature

Univariate Cox regression analysis was used to identify differentially expressed metabolic genes whose expression levels were significantly associated (*P* < 0.05) with overall survival of OS patients in the training data set (TARGET OS). Next, we conducted least absolute shrinkage and selection operator (Lasso) Cox regression analysis to identify metabolic genes related to OS prognosis using the glmnet (Friedman et al., 2010) package, and the OS patients were divided into high- (*n* = 42) and low-risk (*n* = 42) groups based on the median risk score. Finally, we constructed a six metabolic gene signature. To identify the metabolism-related lncRNAs, we performed Pearson correlation analysis between the lncRNAs and metabolic genes related to OS prognosis using | R| ≥ 0.4 and *P* < 0.05 as the selection criteria. Thereafter, we used the same method to screen a metabolism-related lncRNA signature from 147 metabolism-related lncRNAs.

### Evaluation and Verification of the Prognostic Signature

The OS patients were classified into high-risk and low-risk groups based on their prognostic risk score using the median risk score in the metabolic gene signature and metabolism-related lncRNA signature. First, we compared the overall survival of the high-risk and low-risk groups of patients in the two kinds of signatures using the Kaplan–Meier survival curve. According to receiver operating characteristic (ROC) curves, we evaluated the diagnostic efficacy of each clinicopathological characteristic and the prognostic signature for OS patients. Furthermore, univariate and multivariate Cox regression analyses were performed to evaluate whether the risk score was independent of other clinical variables, such as age, gender, and metastasis, in the prognostic signature. We constructed a nomogram by integrating the traditional clinical variables, such as age, gender, and metastasis as well as the risk score derived from the prognostic signature of metabolic genes to analyze the probable 1, 3, and 5-year overall survival of the OS patients. To test whether the prognostic signature of the metabolic genes has a robust ability to predict patient survival in an independent data set, we verified it with an independent data set (GSE21257).

### Gene Set Enrichment Analysis (GSEA) and GSVA

To assess the important functional phenotypes between the high- and low-risk groups based on the eight metabolism-related lncRNA signature, we performed GSEA and GSVA. We used “c5.all.v7.0.symbols.gmt” as the reference gene sets and performed GSEA enrichment analysis using GSEA software (version: 4.0.3). We used “h.all.v7.0.symbols.gmt” as the reference gene set for the GSVA analysis, and the adjusted *p*-value *<*0.05 was considered statistically significant.

### ceRNA Network and Immunization Scores Analysis

Using startbase databases, three of eight metabolism-related lncRNAs were extracted to construct the ceRNA network. We then used three databases (miRTarBase, miRDB, and TargetScan) to search target mRNAs based on the 39 miRNAs. Next, we extracted 88 target mRNAs from the differentially expressed genes. Then, we obtained the immunization scores of immune cells and immune-related functions in the high- and low-risk groups using ssGSEA based on the eight metabolism-related lncRNA signature.

## Results

### Functional Annotation of Differentially Expressed Metabolic Genes

**FIGURE 1:**
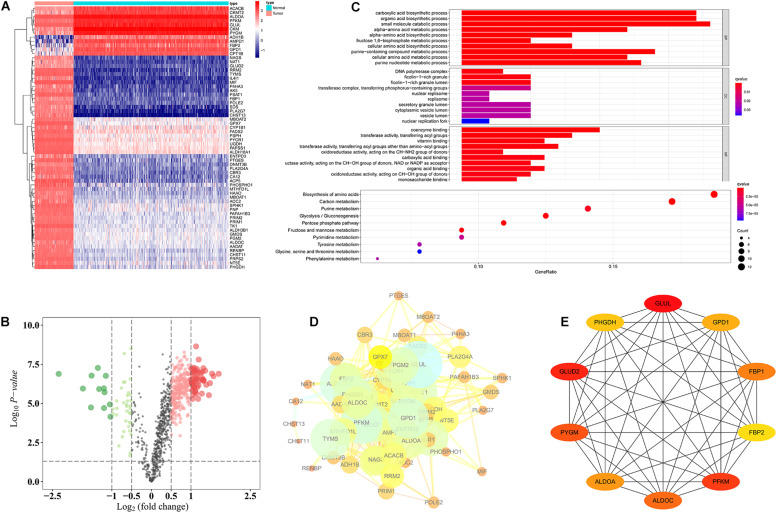
Functional annotation of differentially expressed metabolic genes. **(A)** Heat map of 64 differentially expressed metabolic genes. **(B)** Volcano plot of 944 metabolic genes. **(C)** GO and KEGG analysis of 64 differentially expressed metabolic genes. **(D)** PPI of 64 differentially expressed metabolic genes. **(E)** Ten hub differentially expressed metabolic genes.

First, we used TARGET (*n* = 84) and GTEx (*n* = 396) data sets to compare OS and normal muscle tissues and identified 2,282 differentially expressed genes. We then identified 64 differentially expressed metabolic genes in the 2,282 differentially expressed genes, of which 12 metabolic genes were upregulated in normal tissues, and 52 metabolic genes were upregulated in OS tissues (Figures 1A,B). GO annotation showed that the 64 differentially expressed metabolic genes were involved in various biological functions, including the carboxylic acid biosynthetic process, organic acid biosynthetic process, DNA polymerase complex, ficolin-1-rich granule, coenzyme binding, and transferase activity. KEGG annotation revealed that the 64 differentially expressed metabolic genes were related to signaling pathways, including biosynthesis of amino acids, carbon metabolism, pentose phosphate pathway, and glycolysis/gluconeogenesis (Figure 1C). Last, we constructed a PPI network (Figure 1D) and obtained 10 differentially expressed metabolic genes using Cytoscape: *PHGDH, GLUD2, PYGM, ALDOA, ALDOC, PFKM, FBP2*, *FBP1, GPD1*, and *GLUL* (Figure 1E).

### Identification of Prognostically Significant Metabolic Genes in OS Patients

**FIGURE 2:**
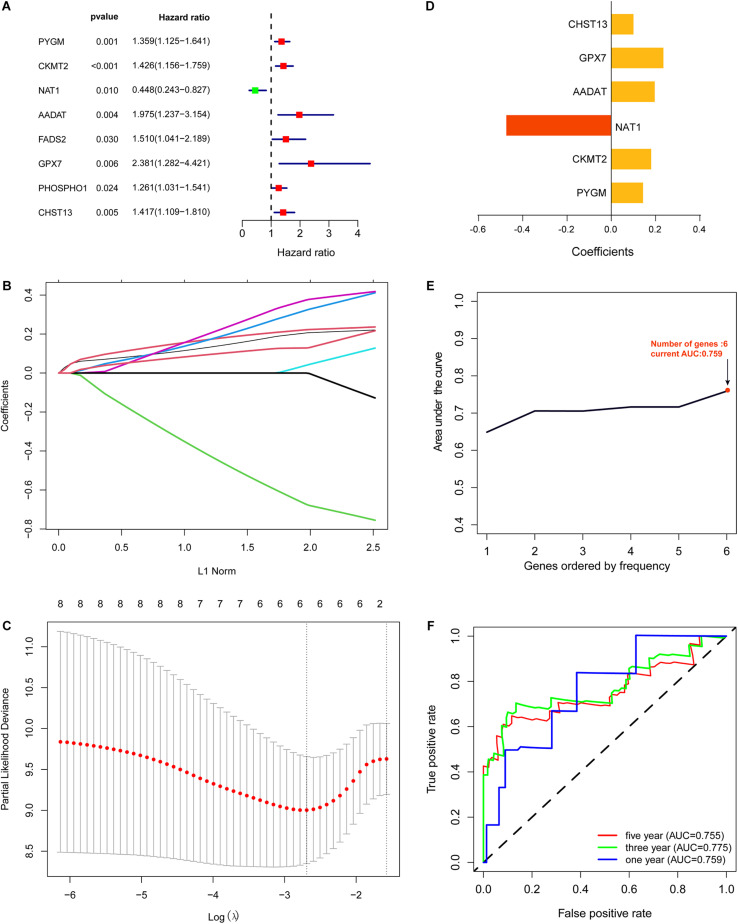
Construction of metabolic gene signature. **(A)** Univariate Cox regression analysis showed that eight out of the 64 differentially expressed metabolic genes significantly correlated with the overall survival of OS patients (*P* < 0.05). **(B,C)** Lasso Cox regression analysis showing that six out of the eight metabolic genes were good candidates for constructing the prognostic signature. **(D)** Coefficients of six out of the eight metabolic genes. **(E)** Six metabolic genes signature constructed by iterative Lasso cox regression analysis. **(F)** ROC curve to evaluate 1, 3, and 5-year prediction efficiency of the six metabolic genes signature.

First, based on the 64 differentially expressed metabolic genes that were screened, we obtained clinical information for the 84 OS samples from the TARGET data set. Then, using univariate Cox regression analysis, we found that eight of the 64 differentially expressed metabolic genes were significantly correlated with overall survival of OS patients (*P* < 0.05; Figure 2A). These genes are the following: *PYGM, CKMT2, NAT1, AADAT, FADS2, GPX7, PHOSPHO1*, and *CHST13*.

### Construction and Evaluation of the Metabolic Genes Signature

To identify a metabolic gene signature for predicting overall survival of OS patients, we conducted Lasso Cox regression for the eight metabolic genes that correlated with the overall survival of OS patients. Lasso Cox regression analysis showed that six of the eight metabolic genes were good candidates for constructing a prognostic signature (Figures 2B–D). Next, we used iterative Lasso Cox regression analysis to construct an optimal prognostic signature of six metabolic genes composed of *PYGM, CKMT2, NAT1, AADAT, GPX7*, and *CHST13* (Figure 2E). ROC analysis indicated that prediction efficiency of the 6 metabolic genes signature was pretty good (1-year AUC = 0.759, 3-year AUC = 0.775, and 5-year AUC = 0.755) and was robust in predicting OS prognosis (Figure 2F).

**FIGURE 3:**
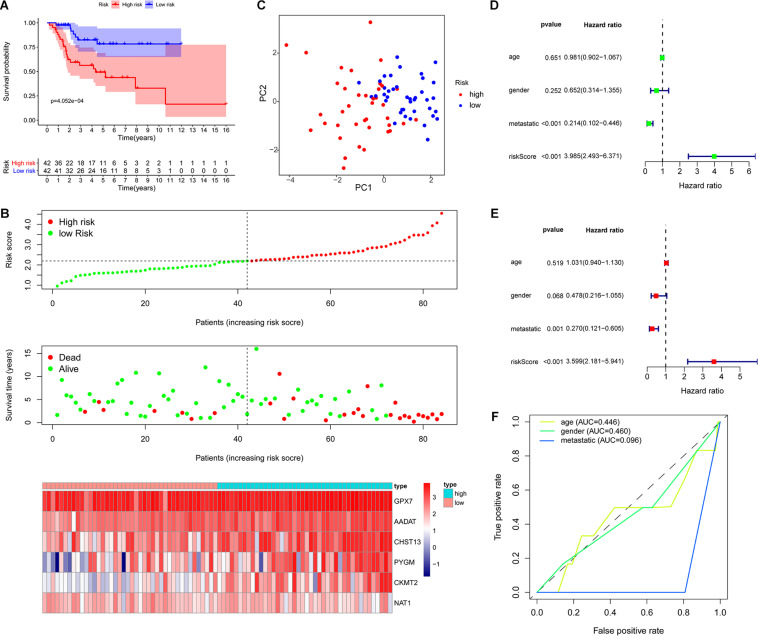
Evaluation of the metabolic gene signature. **(A)** Kaplan–Meier curves of patients in the low-risk and high-risk groups based on the six metabolic genes signature. **(B)** Risk score distribution and survival status of OS patients based on the six metabolic genes signature and heat map of the 6 metabolic genes signature expression pattern. **(C)** PCA based on the confirmed six metabolic genes signature. **(D)** Univariate Cox regression analysis showing that the metastatic and metabolic genes signature risk scores are significantly associated with overall survival. **(E)** Multivariate Cox regression analysis showing that the metabolic genes signature risk score is an independent prognostic indicator for overall survival of OS patients. **(F)** ROC curve analysis with the prognostic accuracy of age, gender, and metastatis.

**FIGURE 4:**
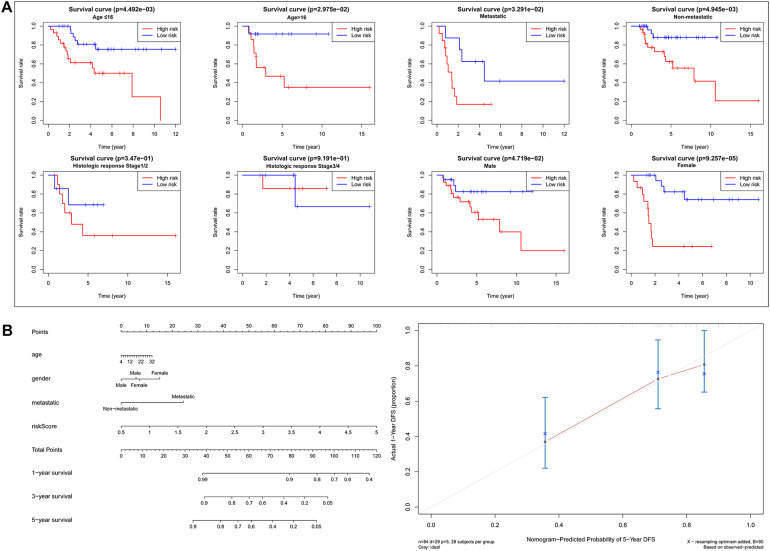
Analysis of prognosis signature with other clinical characteristics. **(A)** Stratification analysis to investigate the prognostic value of the six metabolic genes signature (age, metastatis, gender, histologic response). **(B)** Nomogram for predicting 1, 3, and 5-year survival rates of OS patients based on the six metabolic genes signature risk score and other clinical characteristics, such as age, gender, and metastatis. Calibration curve of the nomogram for 5-year survival rates.

Based on the risk score of each OS patient in the TARGET data set, the patients were divided into high-risk (*n* = 42) and low-risk (*n* = 42) groups. Kaplan–Meier survival curve analysis showed that the overall survival of OS patients with high-risk scores was significantly shorter than those of patients with low-risk scores (Figure 3A). Then, based on the metabolic gene signature, we obtained the risk score distribution, survival status, and a heat map of the six metabolic genes (Figure 3B). Principal component analysis (PCA) based on the six metabolic genes showed two markedly different distribution patterns between high-risk and low-risk groups (Figure 3C). Next, using univariate Cox analyses, we found that the metastatic and metabolic gene prognostic risk scores were significantly associated with overall survival (*P* < 0.001, Figure 3D). Multivariate Cox analyses showed that the prognostic risk score for metabolic genes was significantly associated with overall survival (*P* < 0.001, Figure 3E). As shown in Figure 3F, the ROC curve analysis demonstrated that the AUC values of age, gender, and metastatis were less than the prognostic signature of the metabolic genes (AUC = 0.755). We further performed a stratification analysis to investigate the prognostic value of the metabolic genes (Figure 4A). Finally, we constructed a nomogram and calibration curve analysis to accurately estimate the 1, 3, and 5-year survival probabilities using the risk scores calculated from the metabolic gene prognostic signature and other clinicopathological factors, including age, gender, and metastatis (Figure 4B). These results suggest that the prognosis signature could accurately determine the prognosis of patients with OS.

### Verification of Metabolic Genes Signature in an Independent Cohort

**FIGURE 5:**
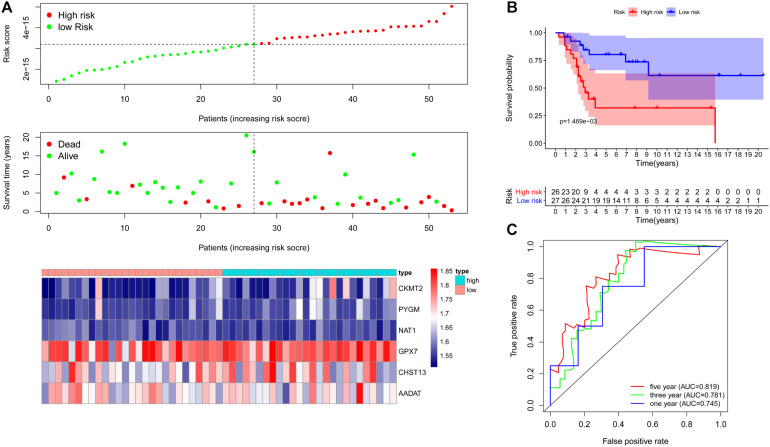
Verification of metabolic gene signature in an independent cohort. **(A)** Risk score distribution and survival status of OS patients based on the six metabolic genes signature and a heat map of the six metabolic genes signature expression pattern in one independent cohort (GSE21257). **(B)** Kaplan–Meier curves of patients in the low- and high-risk groups based on the 6 metabolic genes signature in an independent cohort (GSE21257). **(C)** ROC curve to evaluate 1, 3, and 5-year prediction efficiency of the six metabolic genes signature in an independent cohort (GSE21257).

To further examine the prognostic value of the six metabolic genes, we verified the six metabolic genes signature in an independent cohort (GSE21257). First, based on the risk score for each OS patient in the GSE21257 data set, OS patients were divided into high-risk (*n* = 26) and low-risk (*n* = 27) groups. Kaplan–Meier survival curve analysis showed that the overall survival of OS patients with high-risk scores was significantly shorter than that of patients with low-risk scores (*p* = 1.469e-03, Figure 5A). Next, we obtained the risk score distribution, survival status, and a heat map of the six metabolic genes (Figure 5B) based on the metabolic gene prognosis signature. ROC revealed that the AUCs for 1-, 3-, and 5-year survival were 0.745, 0.781, and 0.819, respectively, in the independent cohort (GSE21257; Figure 5C). These results demonstrate that the six metabolic genes signature can also predict the survival of OS patients in other independent cohorts.

### Identification of Metabolism-Related lncRNAs

To explore metabolism-related lncRNAs, we first identified 148 metabolism-related lncRNAs by performing Pearson correlation analysis between the lncRNAs and the metabolism-related genes using | R| ≥ 0.4 and *P* < 0.05 as the selection criteria. Univariate Cox regression analysis showed that expression of 17 metabolism-related lncRNAs were significantly correlated with the overall survival of OS patients (*P* < 0.05; Table 2).

**TABLE 2:** The 17 metabolism-related prognostic lncRNAs.

lncRNAs	HR	HR.95L	HR.95H	*p*-value
FLJ45513	1.572151	1.107278	2.232194	0.011415
IL10RB-DT	0.554251	0.395923	0.775893	0.000585
TEX41	1.550473	1.0179	2.361693	0.041092
FAM155A-IT1	1.398182	1.04583	1.869246	0.02367
MANCR	0.708946	0.520693	0.965259	0.028924
LINC01517	1.531645	1.239607	1.892485	7.82E-05
LINC02596	1.339465	1.012205	1.772535	0.040871
MSC-AS1	0.651997	0.515729	0.82427	0.00035
LINC00837	1.483295	1.218967	1.804941	8.24E-05
PCED1B-AS1	0.711717	0.530271	0.95525	0.023521
LINC01060	1.381973	1.050937	1.817284	0.020582
CACNA1C-AS1	0.554828	0.383622	0.802442	0.001754
TMEM92-AS1	0.753791	0.602294	0.943395	0.01355
LINC01094	0.676373	0.478496	0.956080	0.026807
LINC02298	2.090637	1.268109	3.446679	0.003838
JMJD1C-AS1	2.090789	1.203725	3.631559	0.00884
LINC00506	0.622487	0.4341	0.89263	0.00995

### Construction and Evaluation of the Metabolism-Related lncRNA Signature

**FIGURE 6:**
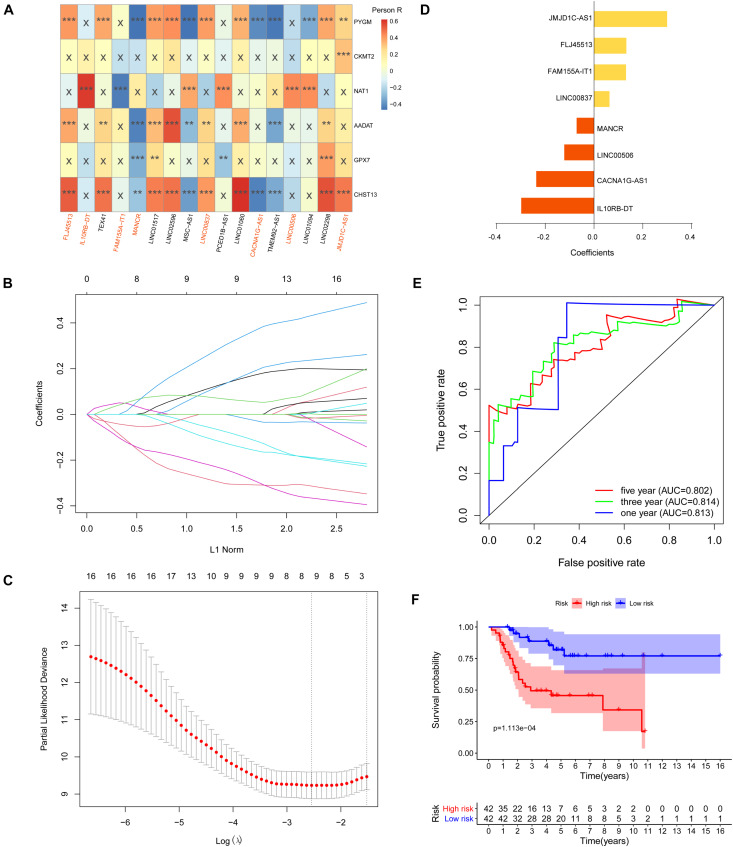
Construction of metabolism-related lncRNA signature. **(A)** Heat map of the correlations between metabolic genes and the 17 prognostic metabolism-related lncRNAs (**p < 0.01 and ***p < 0.001). **(B,C)** Lasso Cox regression analysis showed that eight of the 17 prognostic metabolism-related lncRNAs were good candidates for constructing the prognostic signature. **(D)** Coefficients of the eight metabolism-related lncRNA signature. **(E)** Eight metabolism-related lncRNA signature constructed using iterative Lasso Cox regression analysis. **(F)** ROC curve to evaluate 1, 3, and 5-year prediction efficiency of the eight metabolism-related lncRNA signature.

First, we performed Lasso Cox regression analysis for 17 metabolism-related lncRNAs correlated with the overall survival of OS patients. The analysis showed that eight of the 17 metabolism-related lncRNAs were good candidates for constructing the prognostic signature, including JMJD1C-AS1, FLJ45513, FAM155A-IT1, LINC00837, MANCR, LINC00506, CACNA1C-AS1, and IL10RB-DT (Figures 6A–D). ROC analysis suggested that the metabolism-related lncRNA signature has good performance in predicting overall survival of OS patients, and the AUCs for 1, 3, and 5-year survival were 0.813, 0.814, and 0.802, respectively (Figure 6E).

**FIGURE 7:**
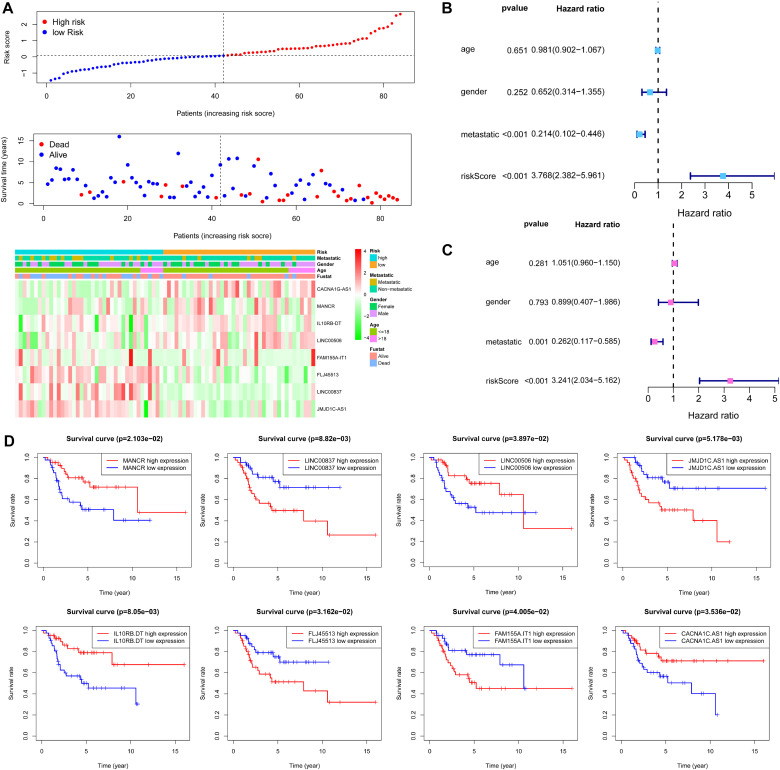
Evaluation of metabolism-related lncRNA signature. **(A)** Risk score distribution and survival status of OS patients based on the eight metabolism-related lncRNAs and a heat map of the eight metabolism-related lncRNAs expression pattern. **(B)** Univariate Cox regression analysis showing the metastatic and the eight metabolism-related lncRNA signature risk score were significantly associated with overall survival. **(C)** Multivariate Cox regression analysis showing that the eight metabolism-related lncRNA signature risk score was an independent prognostic indicator for overall survival of OS patients. **(D)** Kaplan–Meier curves showing that the expression of the eight metabolism-related lncRNA had different overall survival for OS patients.

Based on the risk score of each OS patient in the TARGET data set, OS patients were divided into high-risk (*n* = 42) and low-risk (*n* = 42) groups. Kaplan–Meier survival curve analysis showed that overall survival of OS patients with high-risk scores was significantly shorter than those with low-risk scores (*p* = 1.113e-04, Figure 6F). Based on the eight metabolism-related lncRNAs prognosis signature, we obtained the risk score distribution, survival status, and a heat map of the eight metabolism-related lncRNAs (Figure 7A). Using univariate Cox analyses revealed that the metastatic and metabolism-related lncRNA prognostic risk scores was significantly associated with overall survival (*P* < 0.001; Figure 7B). Multivariate Cox analyses showed that the prognostic risk score of the metabolism-related lncRNAs was significantly associated with overall survival (*P* < 0.001; Figure 7C). Finally, Kaplan–Meier curves showed that patients with different expression levels of the eight metabolism-related lncRNAs had different overall survival (Figure 7D).

### GSEA and GSVA

**FIGURE 8:**
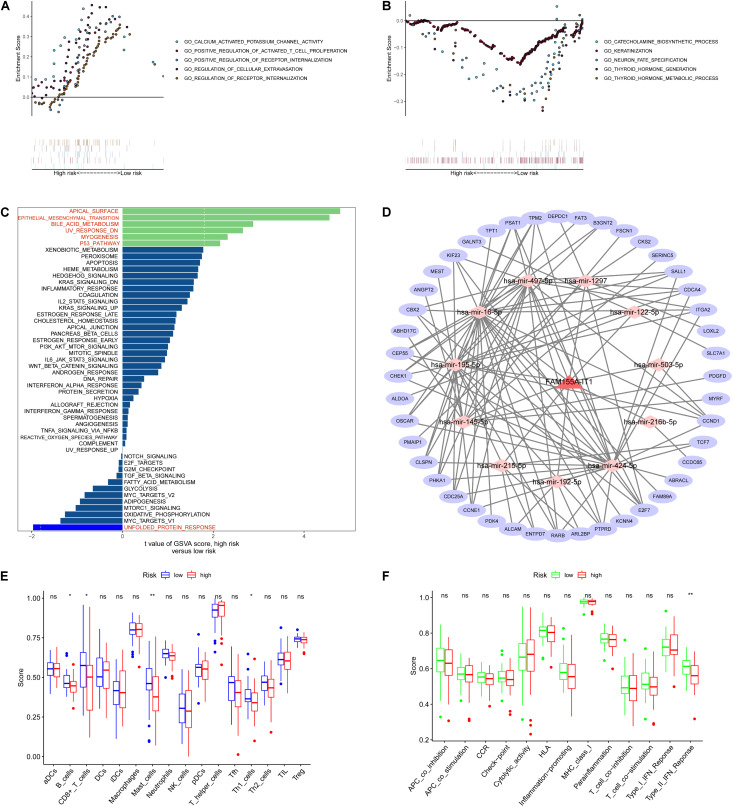
Pathway enrichment, ceRNA network, and immunization scores based on metabolism-related lncRNA signature. **(A,B)** Top five biological processes in the high- and low-risk groups based on GSEA results. **(C)** GSVA results showing that apical surface and bile acid metabolism, epithelial mesenchymal transition, and P53 pathway were activated in high-risk OS patients. **(D)** ceRNA network. **(E,F)** Immunization scores of immune cells and immune-related functions based on the eight metabolism-related lncRNA signature. *p < 0.05, **p < 0.01, ^ns^*p* > 0.05.

To examine the potential biological processes involved, we carried out GSEA based on the eight metabolism-related lncRNA signature. The top five biological processes in the high- and low-risk groups are shown in Figures 8A,B. GSVA results show that apical surface and bile acid metabolism, epithelial mesenchymal transition, and P53 pathway were activated in the high-risk OS patients (Figure 8C).

### Construction of the ceRNA Network and Immunization Score Analysis

To explore the functions of the eight metabolism-related lncRNAs, we constructed a ceRNA network based on the eight metabolism-related lncRNAs (Figure 8D). First, one of the eight metabolism-related lncRNAs were extracted to construct the ceRNA network. We then used three databases (miRTarBase, miRDB, and TargetScan) to search for target mRNAs based on the 11 miRNAs and extracted 44 target mRNAs from differentially expressed genes. Next, we performed immunization scores of immune cells and immune-related functions in the high-risk and low-risk groups using ssGSEA. Results of immunization score analysis show that the immunization scores of B cells, CD8+ T cells, Mast cells, and Th1 cells were significant in the high-risk and low-risk groups (Figure 8E). Furthermore, results of immunization score analysis show that the immunization scores of type II IFN response were significant in the high-risk and low-risk groups (Figure 8F).

## Discussion

In this study, eight metabolic genes were found to be significantly correlated with OS based on univariate Cox regression analysis. Then, 6 metabolic genes (*PYGM, CKMT2, NAT1, AADAT, GPX7*, and *CHST13*) were selected to construct a prognostic signature based on their performance using the Lasso Cox regression analysis. OS patients with high-risk scores showed shorter survival times compared with those with low-risk scores based on the six metabolic genes signature. Univariate and multivariate Cox analyses suggest that the risk score based on the six metabolic gene prognostic signature was an independent prognostic factor. Stratified correlation analysis shows that the metabolic gene signature accurately predicted survival outcomes in high- and low-risk OS patients. In addition, we constructed a nomogram that was good at predicting the 1, 3, and 5-year survival probabilities using the risk score and other clinicopathological factors, including age, gender, and metastatis. Taken together, these results confirm that the six metabolic genes prognostic signature has good performance in predicting the survival outcomes of OS patients in our study.

In recent years, a mounting body of evidence suggests that reprogramming of metabolism in cancer cells has an important effect on cancer development and progression (Hanahan and Weinberg, 2011). Moreover, an increasing number of studies suggests that protein, lipid, and nucleic acid govern cell growth and are activated in cancer cells via tumorigenic mutations, resulting in cancer development and progression (DeBerardinis and Chandel, 2016; Merino Salvador et al., 2017). However, the association between metabolism and OS progression remains unclear. In our study, we identified the six metabolic genes (*PYGM, CKMT2, NAT1, AADAT, GPX7*, and *CHST13*) prognostic signature that showed good performance in predicting survival outcomes of OS patients. However, there are few reports on the role of these genes in OS. *PYGM* is significantly downregulated in head and neck squamous cell carcinoma (HNSCC) and correlates with worse prognosis of HNSCC (Jin and Yang, 2019). In hepatocellular carcinoma, downregulation of *CHST13* regulates the metastasis and chemosensitivity of human hepatocellular carcinoma cells via the mitogen-activated protein kinase (MAPK) pathway (Zhou et al., 2016). Epigenetic inactivation of *GPX7* may be an important mechanism of esophageal cancer (Peng et al., 2009; Peppelenbosch et al., 2014). In Barrett’s esophagus, *GPX7* suppresses bile salt-induced expression of pro-inflammatory cytokines to inhibit Barrett’s carcinogenesis and is also related to gastroesophageal reflux disease–associated Barrett’s carcinogenesis (Peng et al., 2014a, b). From these data, we know that the six metabolic genes identified in our study play different roles in human cancers. However, research regarding the role of these genes is limited in OS. It is, therefore, worth exploring the functions of these six metabolic genes in OS.

Additionally, 17 metabolism-related lncRNAs were found to be significantly correlated with the overall survival of OS patients in this study. Lasso Cox regression analysis showed that eight metabolism-related lncRNAs (JMJD1C-AS1, FLJ45513, FAM155A-IT1, LINC00837, MANCR, LINC00506, CACNA1C-AS1, and IL10RB-DT) were found to be good candidates for the construction of a prognostic signature. Based on the eight metabolism-related lncRNA signature, the clinical outcome of OS patients with high-risk scores were significantly worse than that for patients with low-risk scores. Like the six metabolic genes signature, the eight metabolism-related lncRNAs signature was also independent of other clinical variables, such as age, gender and metastatis. GSVA results showed that bile acid metabolism, epithelial mesenchymal transition, and P53 pathway were activated in high-risk OS patients. Immunization scores analysis suggests that there was a lower score in the high-risk group than in the low-risk group, and high immunity correlated with good prognosis.

Dysregulation of lncRNAs is known to be involved in tumor growth, metabolism, and metastasis (Lin, 2020). Increasing numbers of studies show that dysregulated lncRNAs have an important effect on glucose, lipid, and cholesterol metabolism by regulating mitochondrial function and oxidative stress (van Solingen et al., 2018; Zeng et al., 2018; Lu et al., 2020). Research on the prognostic potential of metabolism-related lncRNAs is limited in OS. Our results suggest that the eight metabolism-related lncRNAs identified in this study can accurately predict overall survival of OS patients. In the eight metabolism-related lncRNAs, MANCR, LINC00837, LINC00506, and IL10RB-DT are relatively well characterized. Tahmouresi et al. (2020) suggests that MANCR is a potential diagnostic biomarker for breast carcinoma (BC) and is associated with aggressive clinical parameters of BC. The study also shows that MANCR was functionally associated with cell proliferation, viability, and genomic stability and represented a potential therapeutic target for BC (Tracy et al., 2018). It is well known that the P53 pathway is related to the metabolism of cancer cells in multiple cancers (Chen et al., 2017; Goyal et al., 2019), and GSVA results also suggest that the P53 pathway was activated in high-risk OS patients. Taken together, these results also suggest that the eight metabolism-related lncRNA signature has strong ability to predict the prognosis of OS patients.

Despite the identification of six metabolic gene and eight metabolism-related lncRNA prognostic signatures, few reports regarding these signatures have been reported in OS previously. In the future, it will be necessary to explore the molecular biological functions of the six metabolic genes and eight metabolism-related lncRNA signatures in OS tumorigenesis and progression, such as in cell proliferation, cell viability, cell metabolism, cell motility, tumor angiogenesis, and drug resistance. In addition, to further validate these metabolic genes and metabolism-related lncRNA signatures in accurate OS diagnosis and prognosis, more clinical evidence, including prospective large-scale cohorts related to these signatures, is crucial. Last, for the potential application of these signatures in the personalized treatment of OS, we believe that it is important to identify the best biomarker and target from these signatures to improve cellular metabolism and immunotherapy of OS. There are several limitations to our study. First, owing to the limited availability of OS sample size (*n* = 84) and clinical data, the subgroup analysis based on other clinical characteristics was limited. Second, the OS sample sizes and clinical data of the independent cohort are also hampered. Last, biological functions of the metabolic genes and metabolism-related lncRNAs need to be verified in the future.

In conclusion, we identify six metabolic gene and eight metabolism-related lncRNA prognostic signatures that show good performance in predicting the survival outcomes of OS patients and are independent of other clinical risk factors. Overall, our study suggests that the six metabolic genes and eight metabolism-related lncRNAs are promising prognostic and diagnostic biomarkers for OS therapy and diagnosis.

## Data Availability Statement

Publicly available datasets were analyzed in this study. This data can be found here: TARGET; https://ocg.cancer.gov/programs/target and Gene Expression Omnibus (GEO; https://www.ncbi.nlm.nih.gov/geo/), GSE21257.

## Author Contributions

NW and ZH-H worked on design and conception of this study. GC-Y, TR, SY-Q, and LT-C collected the data, performed the data analysis, and drafted the manuscript. All authors read and approved the final manuscript.

## Conflict of Interest

The authors declare that the research was conducted in the absence of any commercial or financial relationships that could be construed as a potential conflict of interest.

## Footnotes

1 https://ocg.cancer.gov/programs/target

2 https://www.gtexportal.org/home/

3 https://www.ncbi.nlm.nih.gov/geo/

4 https://www.r-project.org/

5 https://www.string-db.org/
